# Supplementary material for: Leveraging Weighted Quartet Distributions for Enhanced Species Tree Inference from Genome-Wide Data
Source: Genome Biol Evol. 2025 Aug 14;17(9):evaf159. doi: 10.1093/gbe/evaf159 (PMC12401674; doi:10.1093/gbe/evaf159)
Supplement: evaf159_Supplementary_Data [file evaf159_supplementary_data.pdf]

Supplementary material for leveraging weighted quartet distributions for enhanced species tree inference from genome-wide data

Navid Bin Hasan[1], Avijit Biswas[1], Zahin Wahab[1], Mahim Mahbub[1], Rezwana Reaz[1,*], and Md Shamsuzzoha Bayzid[1,*]

[1]Department of Computer Science and Engineering
Bangladesh University of Engineering and Technology
Dhaka-1205, Bangladesh
[*]Corresponding author: rimpi@cse.buet.ac.bd,
shams_bayzid@cse.buet.ac.bd

These supplementary materials present additional results, supplementary figures, and tables.

1 Additional Results

Table S1: Quartet scores of wQFM and wQMC for estimated and true gene trees in different model conditions. The highest scores have been highlighted in bold, and those closest to the true score have been italicized.

**Taxa**	**Model Condition**	**Estimated Gene Trees**			**True Gene Trees**		
		**wQFM-GTF**	**wQMC-GTF**	**True Tree**	**wQFM-GTF**	**wQMC-GTF**	**True Tree**
11	lower-ILS	*40412*	**40421**	40301	50737	***50809***	51252
	higher-ILS	*26680*	**26702**	26372	29268	***29295***	29982
15	100gene-100bp	*69776*	**69930**	69307	***82708***	82045	84634
	100gene-1000bp	*82129*	**82166**	82099	***84437***	84285	84634
	1000gene-100bp	*692949*	**693656**	690268	***834890***	827970	844184
	1000gene-1000bp	*817993*	**818022**	817937	***843791***	843362	844184
37	1X-200-50	*7523379*	**7523568**	7517955	***11707475***	11659108	11744078
	1X-200-250	*10568957*	**10569443**	10565291	***11735692***	11732415	11744078
	1X-200-500	*11271425*	**11271905**	11267990	***11739571***	11738991	11744078
	1X-200-1000	*11586354*	**11586641**	11584969	11743720	***11743809***	11744078
	0.5X-200-500	**10006593**	*10006510*	10003929	***10311452***	10310345	10311622
	2X-200-500	*11947020*	**11947266**	11946371	***12565347***	12565281	12570342
	1X-100-500	*5639605*	**5640131**	5636883	***5871208***	5870844	5874698
	1X-500-500	*28171681*	**28171698**	28171385	***29363114***	29362939	29364013

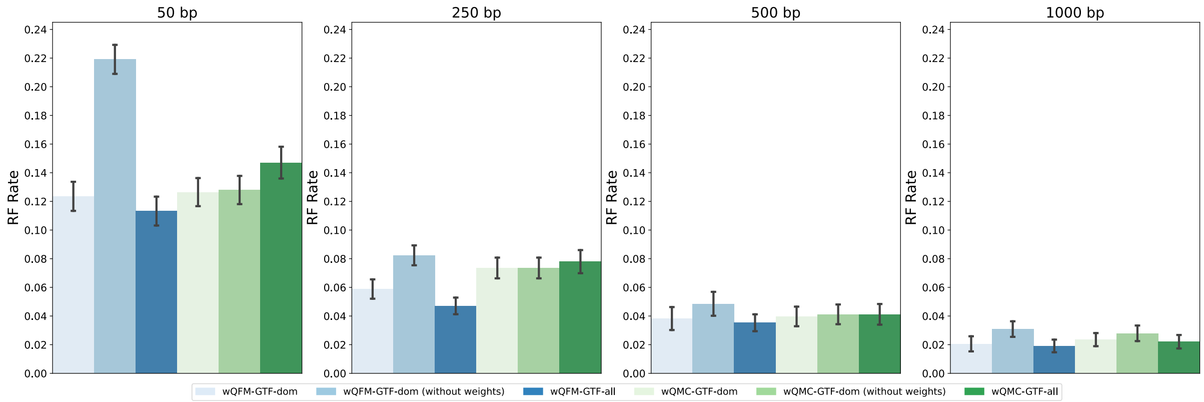

(a) Varying gene tree estimation error

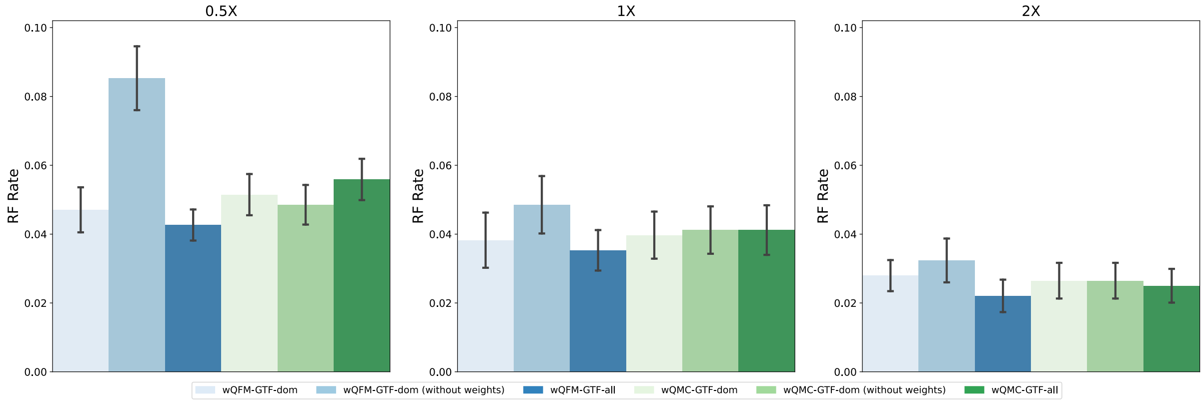

(b) Varying ILS

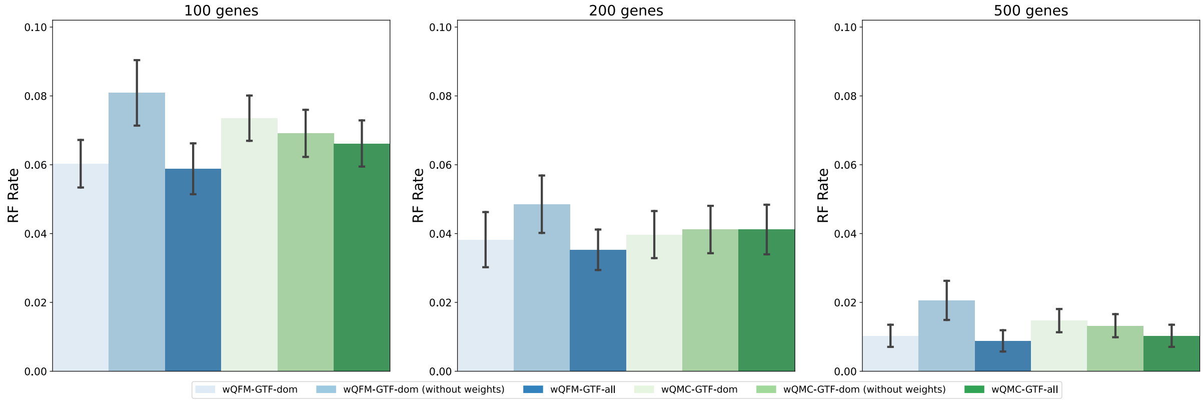

(c) Varying number of genes

Figure S1: **RQ1 (Experiment 1): Results on the 37-taxon dataset.** The quartet distributions are analogous to the 15 taxa version. (a) The sequence length was varied from 50 bp to 1000 bp, keeping the ILS fixed at 1X and the number of genes at 200. (b) The ILS was varied from high (0.5X) to low (2X), keeping the sequence length fixed at 500 bp and the number of genes at 200. (c) The number of genes was varied from 100 to 500, keeping the ILS fixed at 1X and the sequence length at 500 bp.

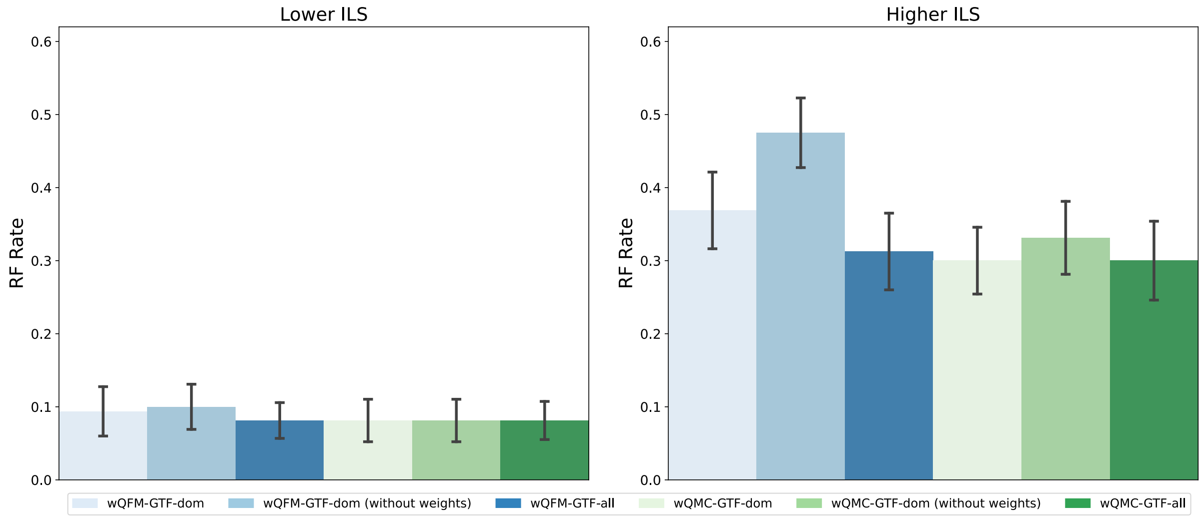

Figure S2: **RQ1 (Experiment 1): Results on the 11-taxon dataset.** The quartet distributions are identical to the 15 taxa version.

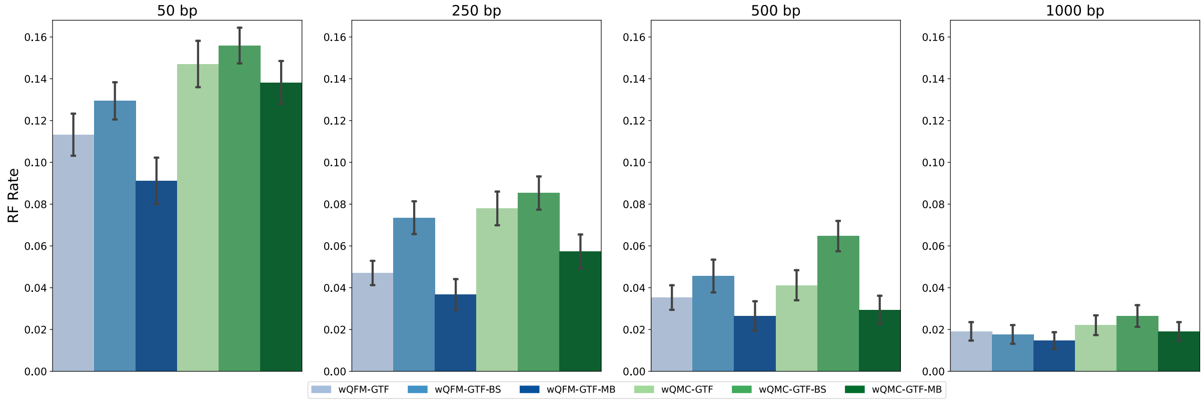

(a) Varying gene tree estimation error

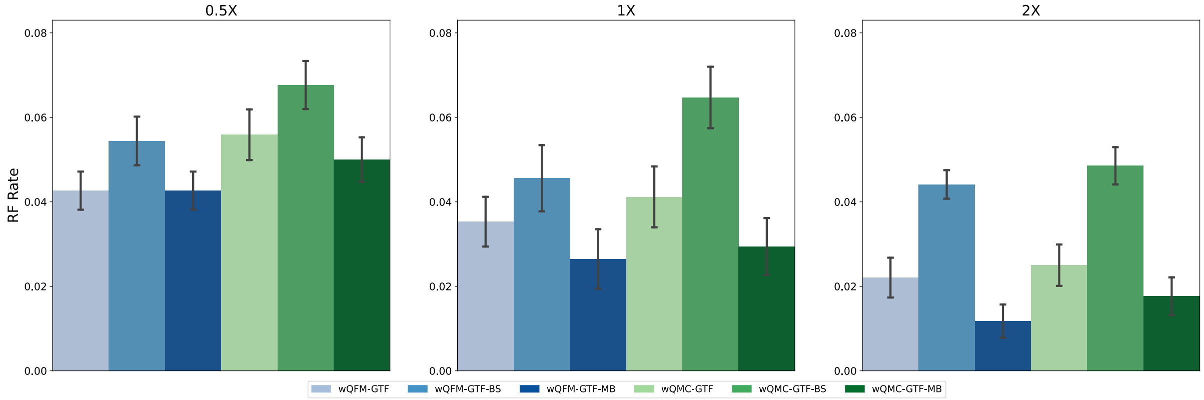

(b) Varying ILS

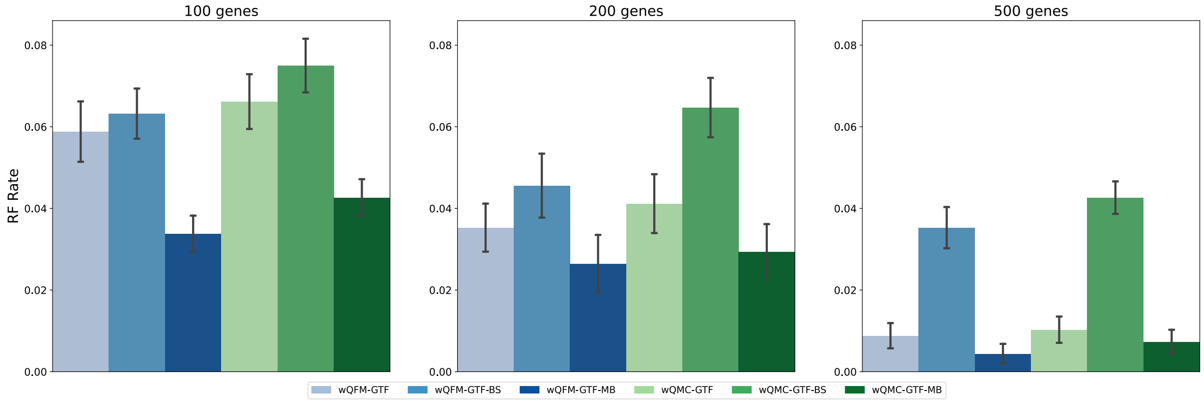

(c) Varying number of genes

Figure S3: **RQ1 (Experiment 2): Results on the 37-taxon dataset.** We compare methods utilizing weighted quartets generated from BestML gene trees (GTF), bootstrap distribution of gene trees (GTF-BS), and Bayesian distribution of gene trees (GTF-MB). (a) The sequence length was varied from 50 bp to 1000 bp, keeping the ILS fixed at 1X and the number of genes at 200. (b) The ILS was varied from high (0.5X) to low (2X), keeping the sequence length fixed at 500 bp and the number of genes at 200. (c) The number of genes was varied from 100 to 500, keeping the ILS fixed at 1X and the sequence length at 500 bp.

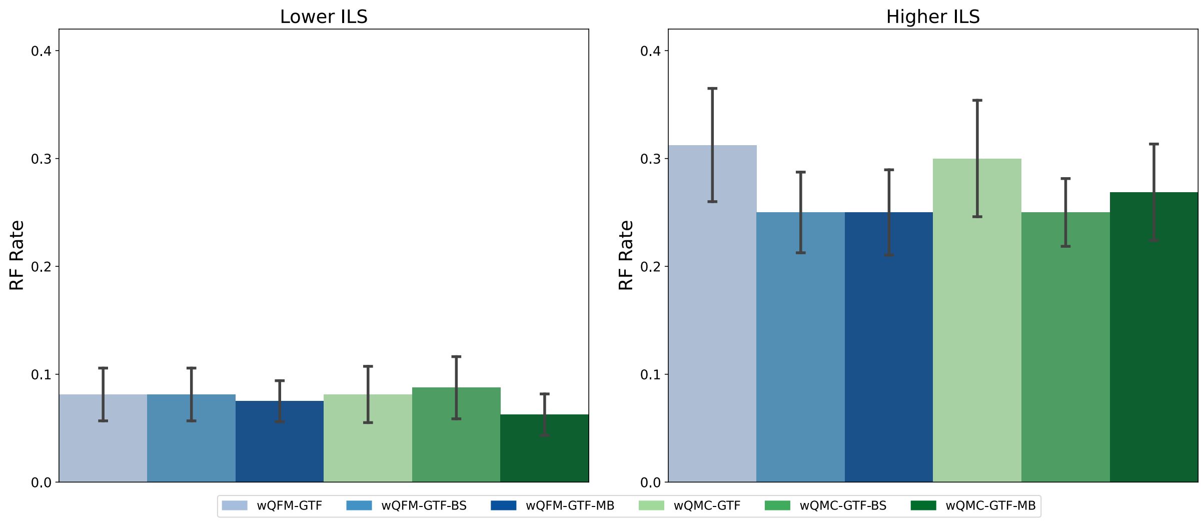

Figure S4: **RQ1 (Experiment 2): Results on the 11-taxon dataset.** We compare methods utilizing weighted quartets generated from BestML gene trees (GTF), bootstrap distribution of gene trees (GTF-BS), and Bayesian distribution of gene trees (GTF-MB).

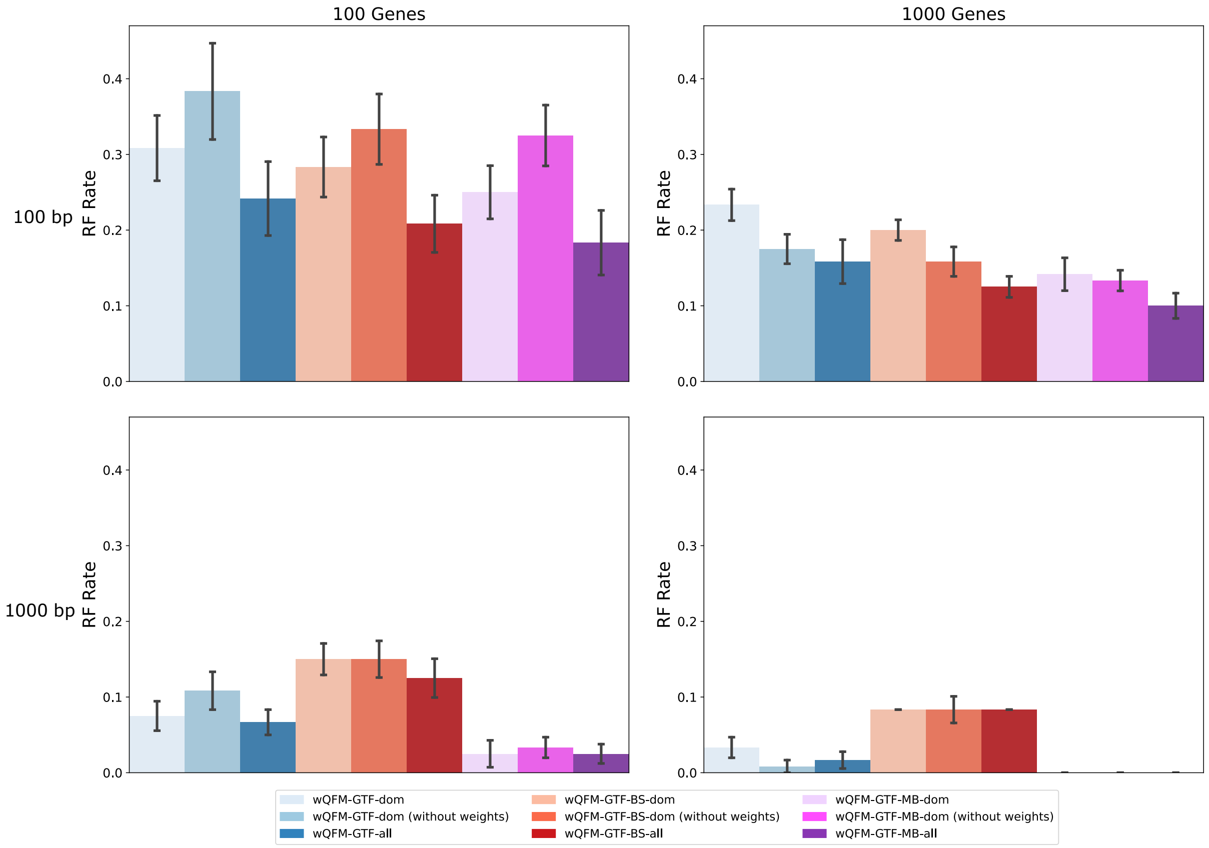

Figure S5: **RQ1: Results on the 15-taxon dataset.** We compare methods utilizing all weighted quartets and only the dominant quartets (with and without weights). Moreover, the quartets were generated from BestML gene trees (GTF), bootstrap distribution of gene trees (GTF-BS), and Bayesian distribution of gene trees (GTF-MB).

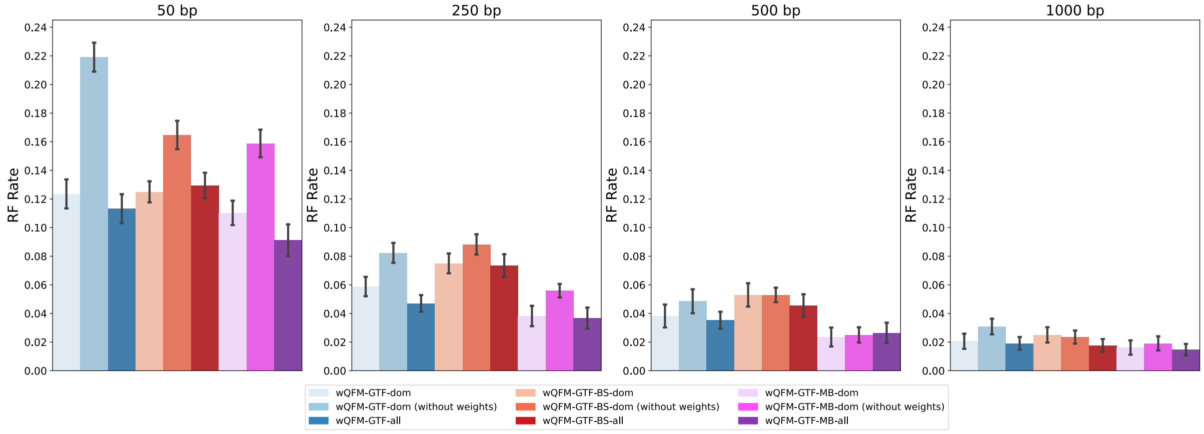

(a) Varying gene tree estimation error

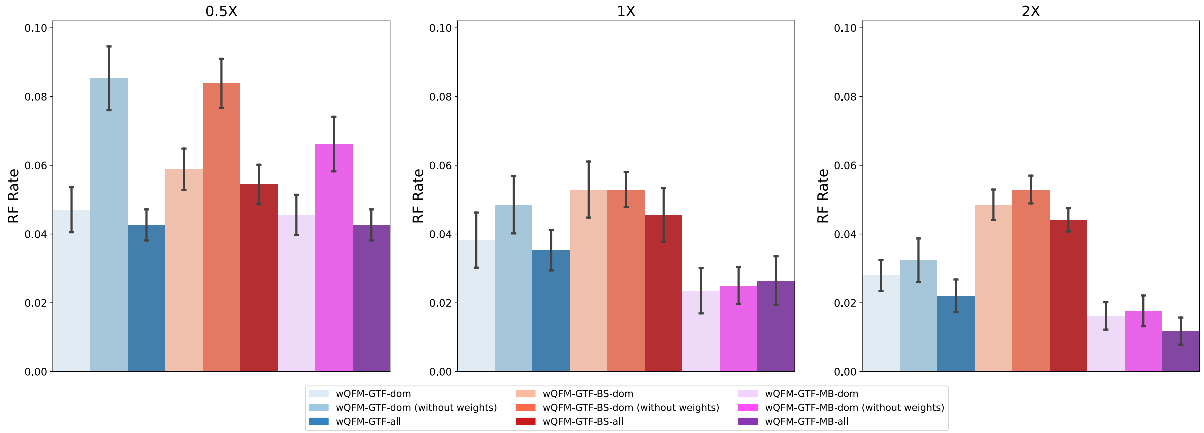

(b) Varying ILS

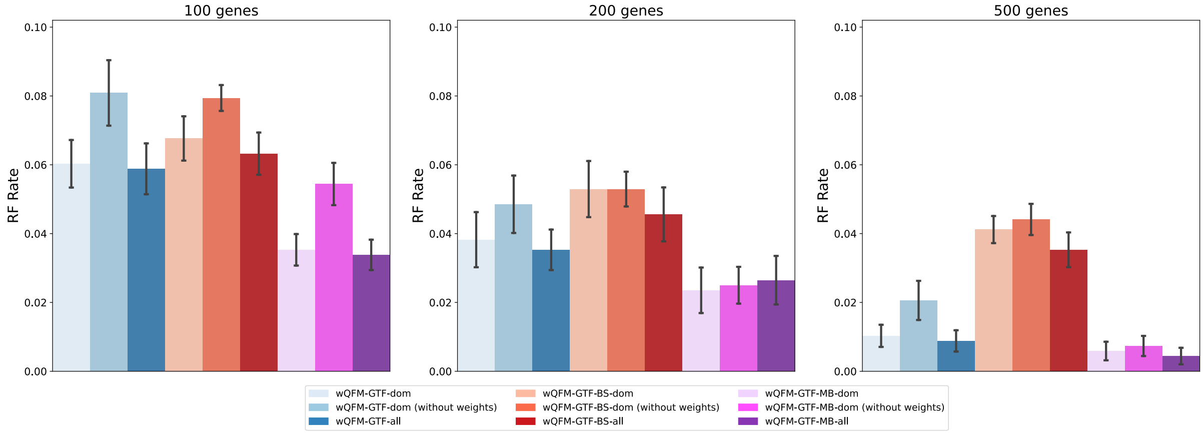

(c) Varying number of genes

Figure S6: **RQ1: Results on the 37-taxon dataset.** We compare methods utilizing all weighted quartets as opposed to utilizing only the dominant quartets (with and without weights). The quartets were generated from BestML gene trees (GTF), bootstrap distribution of gene trees (GTF-BS), and Bayesian distribution of gene trees (GTF-MB).

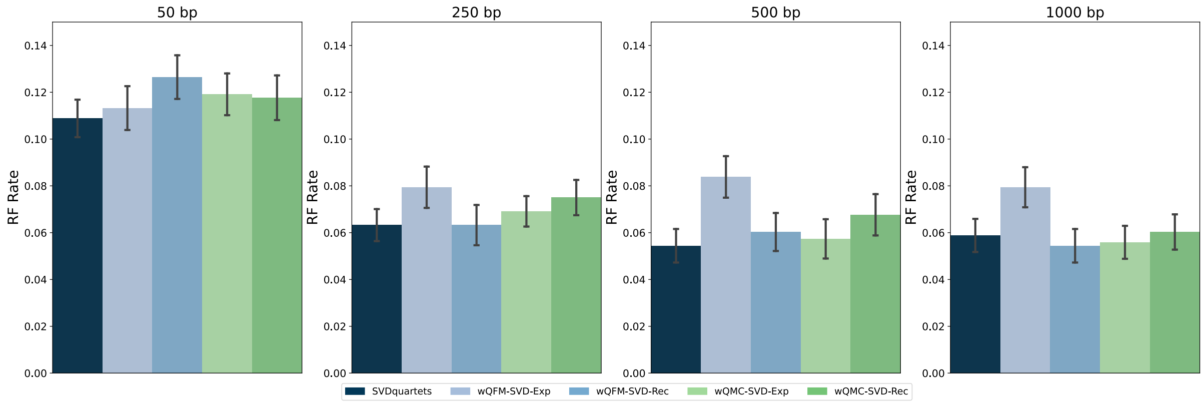

(a) Varying gene tree estimation error

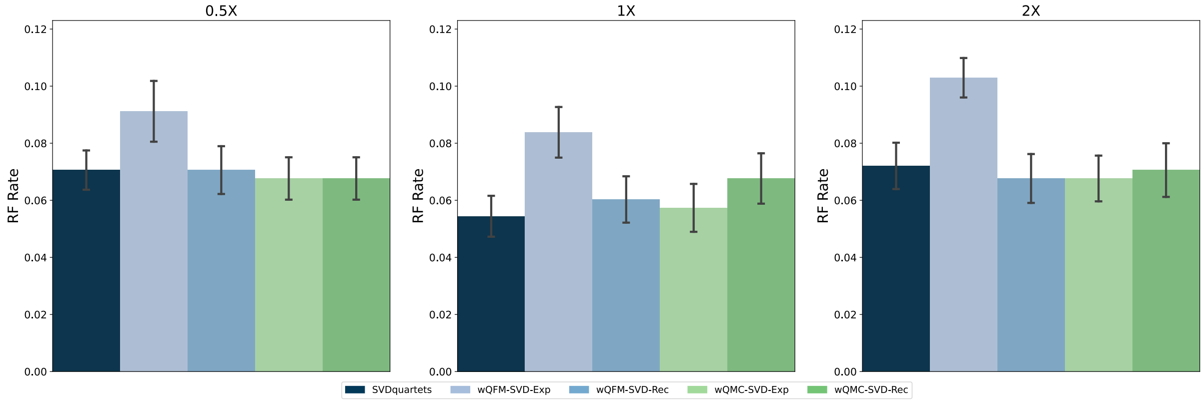

(b) Varying ILS

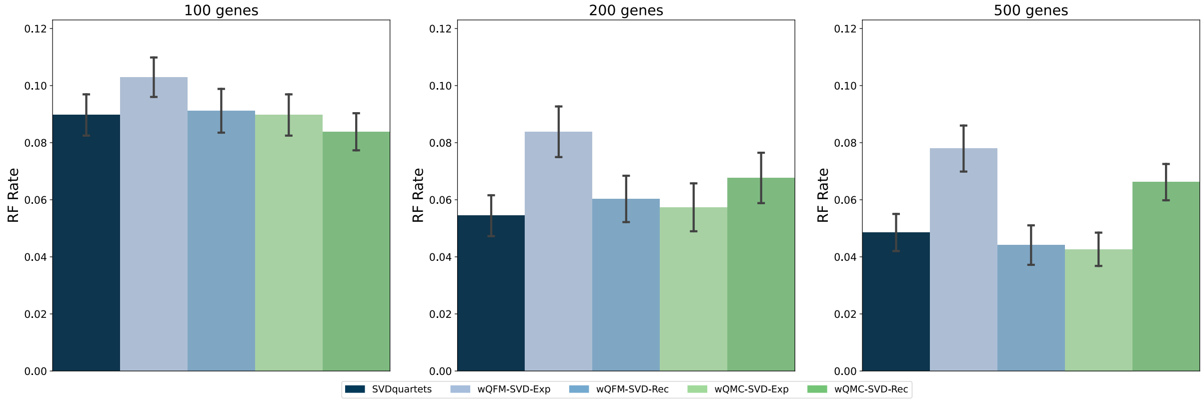

(c) Varying number of genes

Figure S7: **RQ1 (Experiment 3): Results on the 37-taxon dataset.** Performance comparison between unweighted and weighted (both exponential and reciprocal) quartets generated by SVDquartets. The unweighted quartets are amalgamated by QFM, whereas the weighted ones are processed by both wQFM and wQMC. The settings are identical to Figure S3.

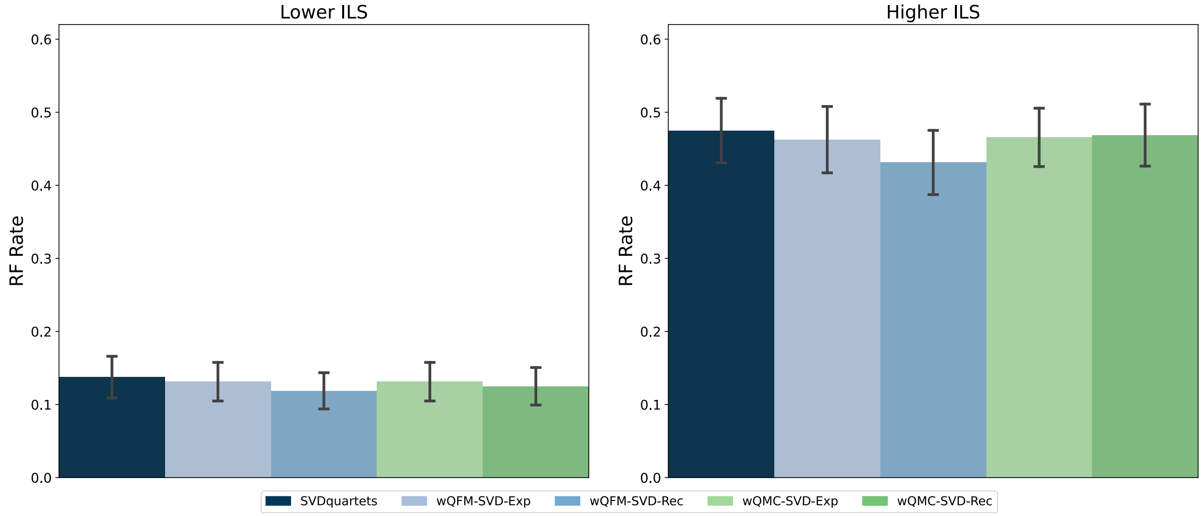

Figure S8: **RQ1 (Experiment 3): Results on the 11-taxon dataset.** Performance comparison between unweighted and weighted (both exponential and reciprocal) quartets generated by SVDquartets. The unweighted quartets are amalgamated by QFM, whereas the weighted ones are processed by both wQFM and wQMC.

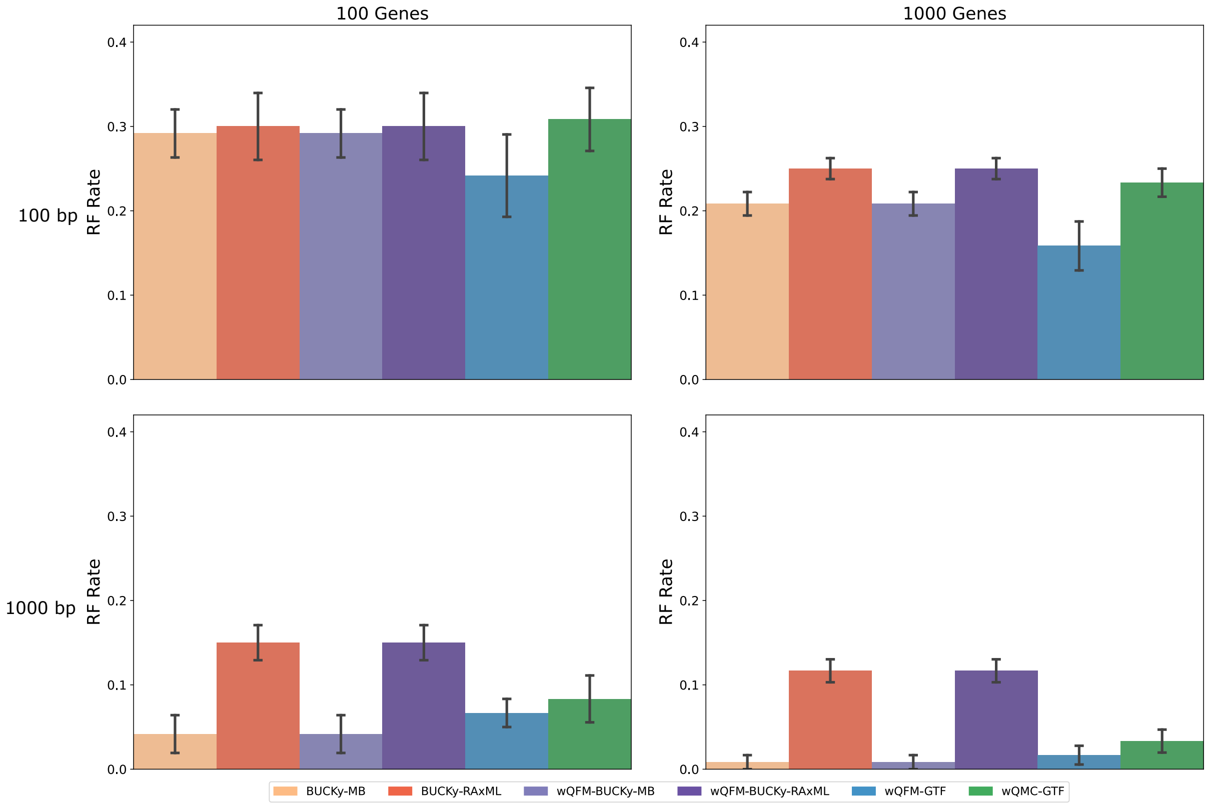

Figure S9: **RQ1 (Experiment 4): Results on the 15-taxon dataset.** Comparison of various BUCKy-based and ML-based methods. We also included wQFM-GTF and wQMC-GTF.

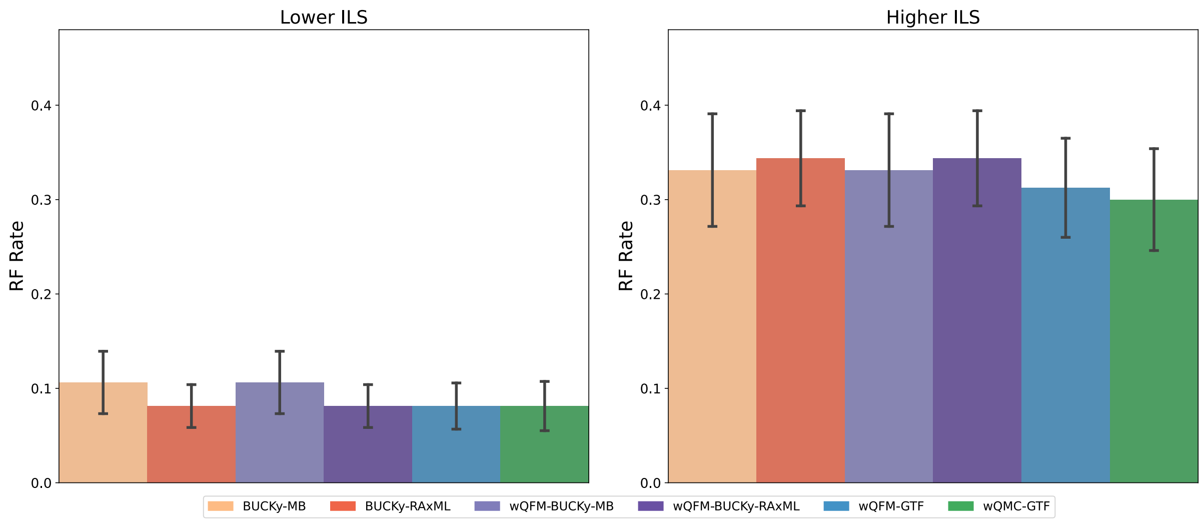

Figure S10: **RQ1 (Experiment 4): Results on the 11-taxon dataset.** Comparison of various BUCKy-based and ML-based methods. We also included the methods wQFM-GTF and wQMC-GTF.

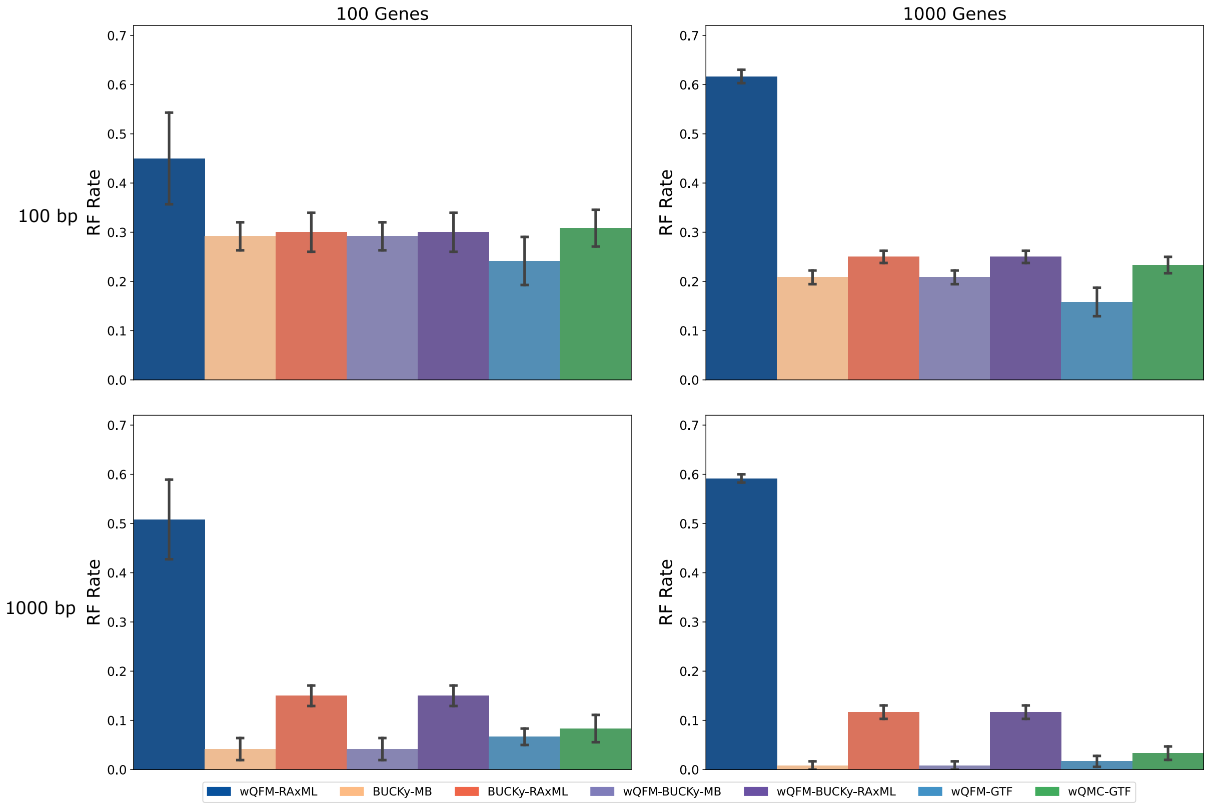

Figure S11: **RQ1 (Experiment 4): Results on the 15-taxon dataset.** Comparison of various BUCKy-based and ML-based methods with wQFM-RAxML added. We also included wQFM-GTF and wQMC-GTF.

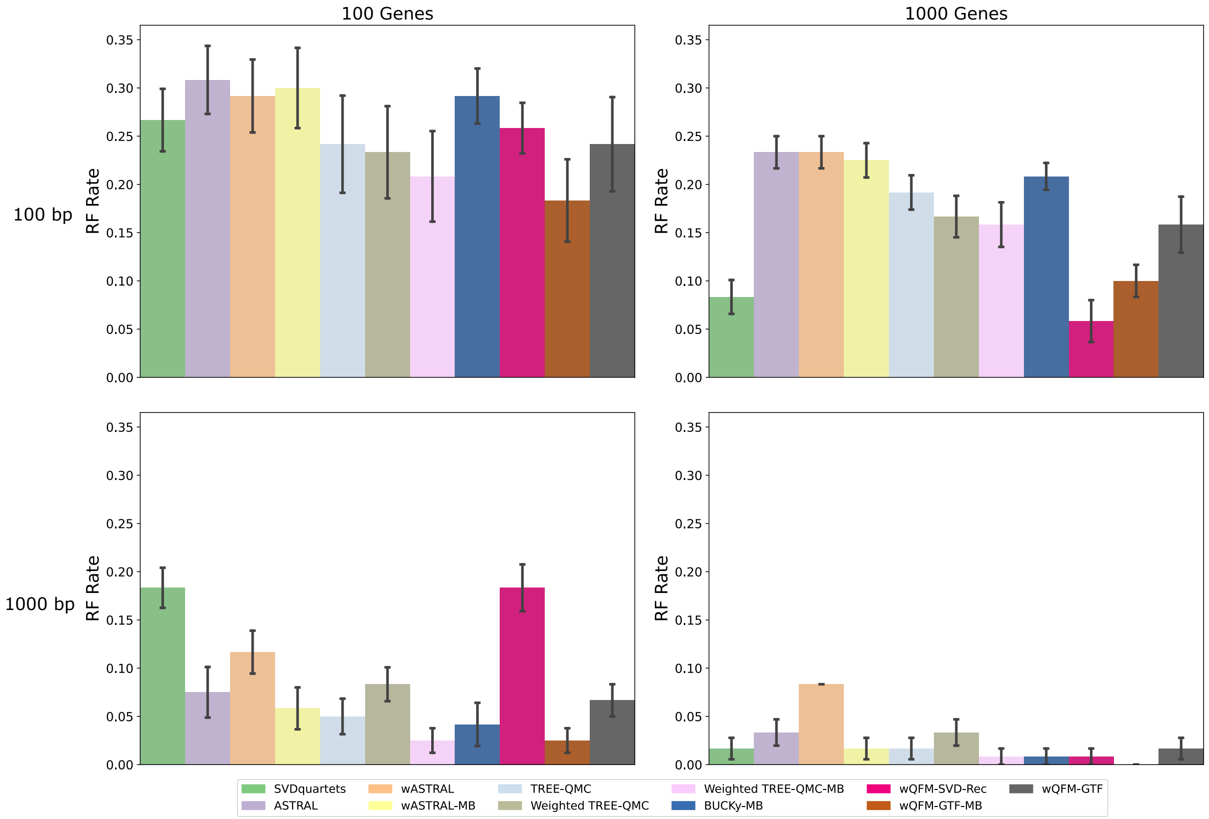

Figure S12: **RQ3: Results on the 15-taxon dataset.** We compare the best methods from previous experiments: wQFM-GTF, wQFM-GTF-MB, wQFM-SVD-Rec with ASTRAL, BUCKy, and SVDquartets. ASTRAL's weighted counterpart, configured to utilize branch supports as weights, was also analyzed. Branch supports were estimated from both non-parametric RAxML bootstrapping (wASTRAL) and Bayesian MCMC sampling (wASTRAL-MB).

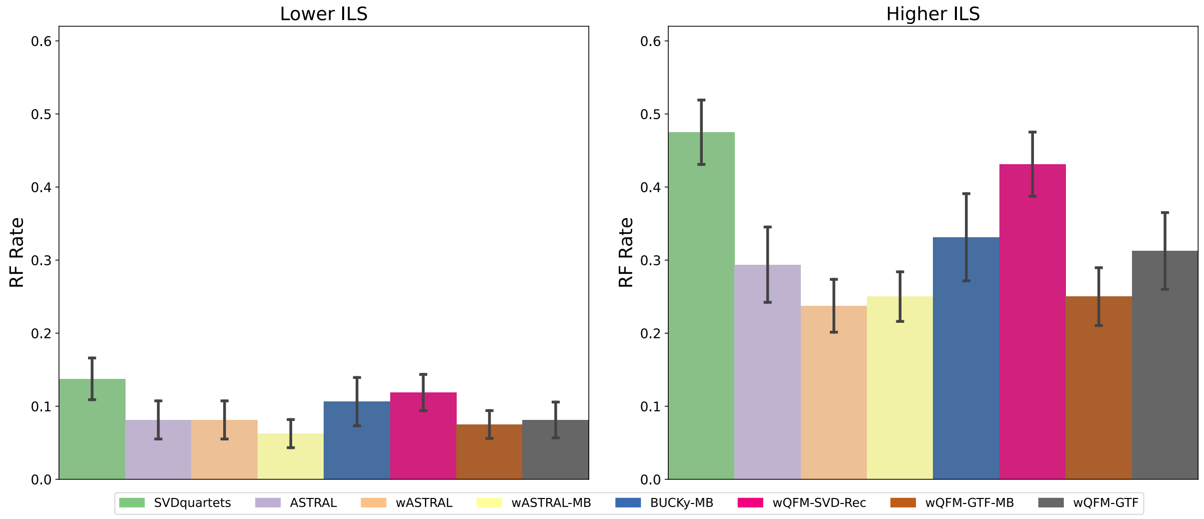

Figure S13: **RQ3: Results on the 11-taxon dataset.** We compare the best methods from previous experiments: wQFM-GTF, wQFM-GTF-MB, wQFM-SVD-Rec with ASTRAL, BUCKy, and SVDquartets. ASTRAL's weighted counterpart, configured to utilize branch supports as weights, was also analyzed. Branch supports were estimated from both non-parametric RAxML bootstrapping (wASTRAL) and Bayesian MCMC sampling (wASTRAL-MB).

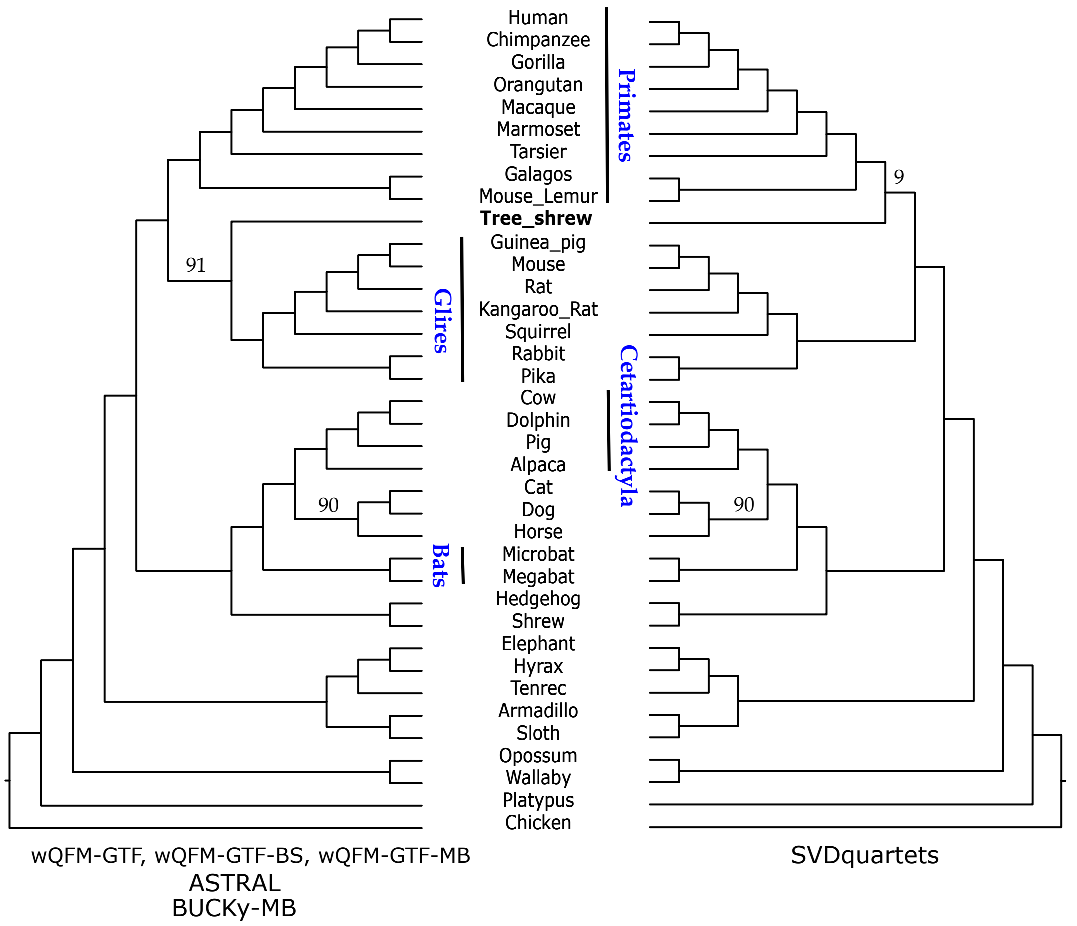

Figure S14: **Analysis of the mammalian dataset.** We show the trees estimated by different methods, e.g., ASTRAL, BUCKy, SVDquartets, and wQFM with different types of quartet distributions. The values on the branches of each tree denote their corresponding support values. Branch supports are computed based on quartet-based local posterior probability (multiplied by 100). All BS values are 100% except where noted.
